# Assessing Curcumin Uptake and Clearance and Their Influence on Superoxide Dismutase Activity in *Drosophila melanogaster*

**DOI:** 10.3390/biotech12030058

**Published:** 2023-09-08

**Authors:** Tammy R. Hoffman, Sarah A. Emsley, Jenna C. Douglas, Kaela R. Reed, Abigail R. Esquivel, Marc J. Koyack, Brie E. Paddock, Patrick Videau

**Affiliations:** 1 Department of Biology, Southern Oregon University, Ashland, OR 97520, USA; 2 Department of Chemistry, Southern Oregon University, Ashland, OR 97520, USA; 3 Department of Molecular Medicine, Morsani College of Medicine, University of South Florida, Tampa, FL 33602, USA; 4 School of Arts and Sciences, Gwynedd Mercy University, Gwynedd Valley, PA 19437, USA

**Keywords:** *Drosophila*, curcumin, superoxide dismutase

## Abstract

While normal levels of reactive oxygen and nitrogen species (RONS) are required for proper organismal function, increased levels result in oxidative stress. Oxidative stress may be managed via the scavenging activities of antioxidants (e.g., curcumin) and the action of enzymes, including superoxide dismutase (SOD). In this work, the uptake and clearance of dietary curcuminoids (consisting of curcumin, demethoxycurcumin, and bisdemethoxycurcumin) was assessed in *Drosophila melanogaster* larvae following chronic or acute exposure. High levels of curcuminoid uptake and loss were observed within a few hours and leveled off within eight hours post treatment onset. The addition or removal of curcuminoids from media resulted in corresponding changes in SOD activity, and the involvement of each of the three *SOD* genes was assessed for their contribution to total SOD activity. Taken together, these data provide insight into the uptake and clearance dynamics of curcuminoids and indicate that, while SOD activity generally increases following curcuminoid treatment, the individual *SOD* genes appear to contribute differently to this response.

## 1. Introduction

Oxidative stress, the imbalance between reactive oxygen and nitrogen species (RONS) and endogenous antioxidant systems, has been implicated in disease [1]. RONS are a group of short lived and highly reactive species, including superoxide (O_2_^−^), peroxide (OH^−^), nitrogen dioxide (•NO_2_), and others. Although normal RONS levels are necessary to maintain proper signaling and physiological functions, excessive amounts can lead to the denaturation and degradation of proteins and DNA, lipid peroxidation, toxicity, and cell death [2,3]. As research has indicated oxidative stress may be responsible for initiating and accelerating chronic and degenerative disease and aging, it is crucial to identify potential therapeutic compounds that directly target and reduce excessive RONS to physiological levels. In recent years, curcumin, a yellow phenolic compound derived from the *Curcuma longa* plant, has been studied for its multiple bioactivities, including its function as a potent antioxidant [4,5,6]. While it is speculated that the polyphenolic rings in curcumin may directly scavenge RONS, curcumin may also act as a signaling molecule that influences the expression and/or activity of enzymes involved in reducing oxidative stress and its effects. Although the mechanism by which curcumin acts remains under investigation, research indicates that curcumin treatment results in extensive changes to gene expression, specifically in genes related to endogenous antioxidants [5,7,8,9,10]. These results suggest that curcumin may be acting, at least in part, via modulation of specific protective genetic elements.

Previous studies have shown that curcumin treatment results in an increase in superoxide dismutase (SOD) activity, an enzyme that catalyzes the conversion of superoxide into oxygen (O_2_) and hydrogen peroxide (H_2_O_2_) [2]. SOD exists in three different forms encoded by separate genes. While these forms have functional similarities, they are distinct in sequence, structure, cofactor requirements, and cellular compartmentalization [2,11]. SOD1 exists almost exclusively as a cytosolic 88 kDa homodimer that requires copper and zinc cofactors to function [12]. In contrast, SOD2 is a 32kDa homotetramer existing exclusively in the mitochondria, and has a manganese cofactor. Lastly, SOD3 is a 135kDa tetramer that primarily functions extracellularly, and utilizes copper and zinc cofactors. Changes in SOD activity and expression have also been linked to disease [2,13]. SOD has multiple genetic links to amyotrophic lateral sclerosis (ALS), a debilitating neurological disease [11,14,15], as well as links to chronic kidney disease, cancer progression, and chronic obstructive pulmonary disease (COPD) [16,17,18]. Disease intervention by targeting or mimicking elements within the antioxidant systems, such as SOD, has led to promising results in recent studies. In one study, the ingestion of cerium oxide nanoparticles (nCeO_2_) correlated with the restoration of SOD levels in a transgenic *Drosophila melanogaster* Alzheimer’s disease model [19]. As *Drosophila* is an accessible model system to study the molecular underpinnings of disease, strides have been taken to study SOD in this model to provide the basis for developing future therapies [20,21].

Previous studies have indicated a link between SOD expression and/or activity and responses to curcumin treatment. *Drosophila* fed a curcumin-supplemented diet were found to have an increased lifespan, which co-occurred with increased SOD activity and upregulation of SOD gene expression [9]. Similarly, feeding *Drosophila* larvae curcumin increased SOD and acetylcholinesterase activities while promoting an increased lifespan [22]. Under heat stress conditions, feeding curcumin to *Drosophila* increased lifespan and SOD gene expression [23]. The addition of disulfiram, an SOD inhibitor, negated the lifespan increases associated with curcumin-supplemented diets in *Drosophila* [24]. Curcumin treatment also mitigated both copper-induced oxidative stress and increased aging rates following irradiation in *Drosophila*, and oxidative stress in astrocytes [25,26,27]. Interestingly, in the aforementioned studies that identified increased SOD activity following curcumin treatment, only total SOD activity was assessed, and the publications did not distinguish between activities originating from individual SOD homologs.

We previously found that curcumin treatment in *Drosophila melanogaster* lowered the oxidative stress burden, increased lifespan, and affected behavior in a sex-dependent manner [28]. It was found that, when reared on several concentrations of curcuminoids, the larvae accumulated curcuminoids in their tissues and this was facilely quantified via methanol extraction and HPLC analysis. Additionally, several sex-specific outcomes were observed: increases in longevity and decreases in oxidative stress in females, as well as motility issues in males. While formative, this study focused on phenotypic sex differences and did not elucidate a specific mechanism underlying the decreased oxidative stress levels observed. Given the differences among SOD homologs, understanding how curcumin may influence each will begin to define a mechanism by which curcumin may act to modulate oxidative stress. Here, we assess the uptake and clearance of dietary curcumin in *Drosophila* and determine its influence on modulating isoform-specific SOD activity.

## 2. Materials and Methods

### 2.1. Drosophila Husbandry and Curcuminoid Treatments

All *Drosophila melanogaster* were maintained as previously described [28]. Briefly, all strains were maintained on Nutri-Fly German formulation medium (GF; Genessee Scientific, San Diego, CA, USA) at 25 °C in a 12 h:12 h light:dark cycle at 60% humidity. The Oregon R strain is denoted as wild type (WT) in this work. The SOD1, SOD2, and SOD3 deficiency lines utilized, Sod1[X-39] e[1]/TM3, Sb[1] Ser[1] (#24490), Df(2R)Exel7145 (#7887), and y[1] w[67c23] (#14138), respectively, were obtained from the Bloomington Stock Center and are referred to as SOD1, SOD2, and SOD3 throughout. These were chosen because the individual SOD mutations each line harbors have been the focus of previous studies and represent useful lines for assessment [29,30,31]. Curcuminoid treatments were prepared with the desired concentration of curcuminoids (0–25 mM final concentrations in GF medium), derived from *Curcuma longa* (C1386, Sigma-Aldrich, St Louis, MO, USA; 98% pure standard consisting of curcumin, demethoxycurcumin, and bisdemethoxycurcumin in a 67:29:4 ratio and referred to as curcuminoids hereafter) as previously described [28].

### 2.2. Larval Preparation, Extraction, and HPLC Analysis

Groups of 10 third-instar larvae were collected and extracted as previously described [28]. Larvae reared on GF medium or GF supplemented with 25 mM curcuminoids were rinsed in 1× PBS and blotted dry before transfer onto a new medium for the durations stated. HPLC analysis of curcuminoid levels in larvae was conducted as previously described [28]. To assess curcuminoid levels that could enter an SOD assay (described below), larvae were extracted with extraction buffer (10 mM Tris-base pH 7.4) and the rest of the protocol and analysis remained unchanged.

### 2.3. Larval Preparation and SOD Assay

Larvae were reared from hatching on GF medium or GF supplemented with 25 mM curcuminoids, and were rinsed in 1× PBS and blotted dry before use. All manipulations were conducted on ice within 30 min of harvest. The 1 h PBS incubation conducted prior to extraction for HPLC analysis was omitted to ensure that the SOD activity levels measured were not altered. After rinsing, 250 μL of extraction buffer (10 mM Tris-base pH 7.4) was added to groups of 5 larvae, which were ground for roughly 1 min using a motorized Kontes pellet pestle rod system and then sonicated on ice using a Qsonica Q125 sonicator (Melville, NY, USA) with a 0.25-inch probe for one pulse of 10 s at 40% power. Extracts were centrifuged at 14,000× *g* for 10 min and the supernatant was transferred to a fresh tube, taking care not to disturb the pellet. Protein concentrations were determined via Bradford assay using the Bio-Rad Protein Assay Kit II according to the manufacturer’s instructions (Bio-Rad, Hercules, CA, USA). SOD activity was quantified using the Superoxide Dismutase (SOD) Activity Assay Kit according to the manufacturer’s instructions (#CS0009; Sigma-Aldrich, St. Louis, MO, USA). Activity was measured in technical duplicate for each biological replicate, and the results were averaged from the means of ten biological replicates per treatment.

### 2.4. Analyses

Significance in curcuminoid concentrations and SOD activities between conditions was assessed via student’s *t*-test and an alpha value of 0.05, which were conducted with the Microsoft Excel software (Microsoft Corporation, Redmond, WA, USA). Graphical preparation of data was conducted using the GraphPad Prism software (GraphPad, San Diego, CA, USA).

## 3. Results

### 3.1. Assessing Uptake and Clearance Parameters of Curcuminoids in Drosophila Larvae

**Figure 1:**
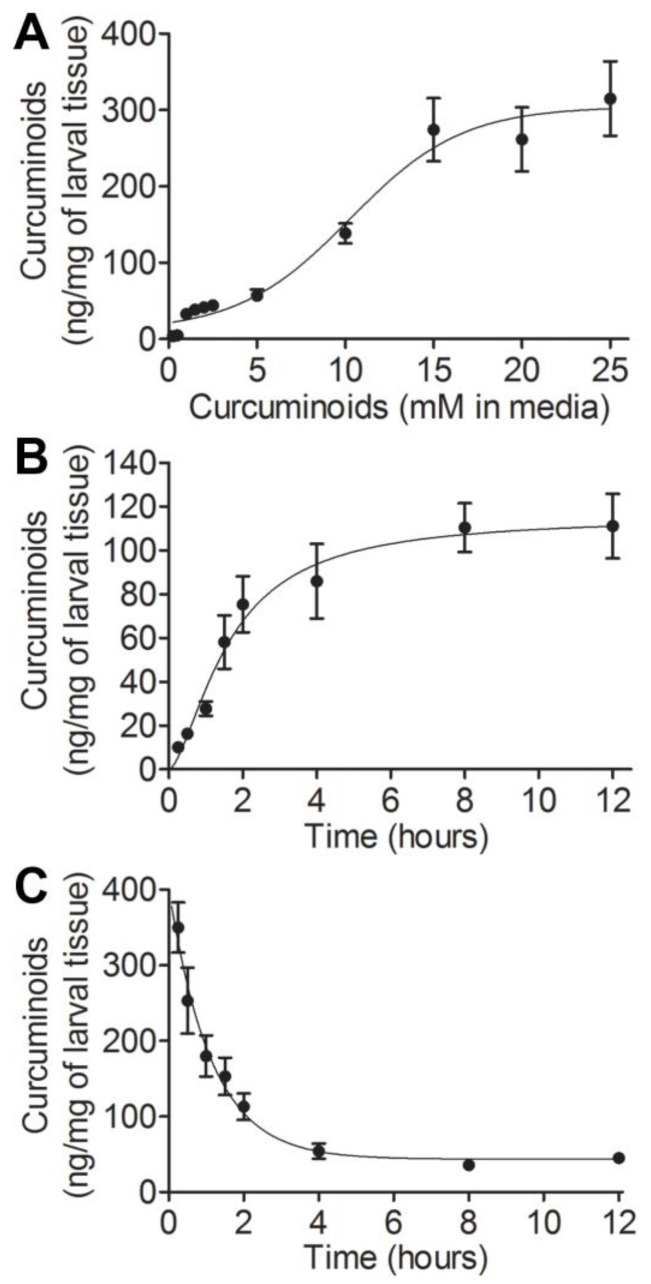
Determination of the influence of time and concentration on curcuminoid uptake in wild type (WT) larvae. Larvae were reared for seven days and the level of curcuminoids in tissues was quantified from groups of 10 crawling third-instar larvae (*n* = 10 per treatment). Larvae were reared on varying curcuminoid concentrations (**A**) on 0 mM curcuminoids and transferred to 25 mM curcuminoids (**B**), or on 25 mM curcuminoids and transferred to 0 mM curcuminoids (**C**), which resulted in the following equations of best fit: $y=10.23+\frac{294.17}{1+10^{10.26-x}}$, (r^2^ = 0.7382); $y=\frac{115.4*X^{1.608}}{2.151+X^{1.608}},$ (r^2^ = 0.7650); and $y=359.38^{-0.885*X}+43.42$, (r^2^ = 0.7562), respectively. The mean of experiments conducted in *n* = 10 is presented, and error bars represent the standard error of the mean.

While our previous study demonstrated quantitative methodology and chemical, developmental, and behavioral changes that were altered in crawling third-instar WT *Drosophila melanogaster* larvae following curcuminoid (curcumin and its derivatives demethoxycurucmin and bisdemethoxycurcumin) treatment, additional relevant parameters remain undefined in this model system [28]. Larvae reared on either 0.25, 2.5, or 25 mM curcuminoids retained different levels of curcuminoids, but the influence of curcuminoid concentration on tissue uptake and retention is unknown. To address this, *Drosophila* larvae were reared from hatching on various curcuminoid concentrations (a chronic treatment) and the level of curcuminoids in their tissues was quantified in seven-day wandering third-instar larvae. The level of curcuminoids in larval tissue increased concomitant with curcuminoid concentration in the medium and began to plateau by 15 mM curcuminoids in media (Figure 1A). The average curcuminoid concentrations in tissues of larvae reared on 15, 20, and 25 mM curcuminoids were 274 ± 44, 262 ± 45, and 315 ± 52 ng/mg of tissue, respectively, which were not significantly different from one another (paired *t*-tests, each *p* > 0.41).

The results presented above provide insight into the bounds of curcuminoid carrying capacity as a result of chronic exposure to curcuminoids but do not describe the time-dependence of curcuminoid uptake or loss (an acute exposure). To address this, larvae reared on 0 or 25 mM curcuminoids were transferred to the opposite condition (i.e., larvae reared on 0 mM were transferred to 25 mM curcuminoids and vice versa) and curcuminoids in their tissues were quantified over the next 12 h. Curcuminoid uptake was quick in the first two hours immediately following larval transfer onto media supplemented with 25 mM curcuminoids, and began to stabilize roughly four hours post transfer (Figure 1B). The highest concentration of curcuminoids reached after 12 h of exposure was 111 ± 16 ng/mg of tissue, which is significantly lower than the concentration reached after seven days of chronic exposure (315 ± 52 ng/mg of tissue; *t*-test, *p* = 0.003). Similarly, the transition from 25 mM to 0 mM curcuminoids saw a swift drop in curcuminoid concentration in the tissues, which began to level off about four hours post transfer (Figure 1C). After 12 h on medium lacking curcuminoids, the larvae maintained 45 ± 7 ng/mg of tissue, which is similar to the level seen after seven days on medium supplemented with 5 mM curcuminoids (57 ± 9 ng/mg of tissue; *t*-test, *p* = 0.27). Taken together, these results indicate that curcuminoids display uptake and clearance within hours of treatment onset and that curcuminoid levels are retained and cleared differently between acute and chronic exposures.

### 3.2. SOD Levels Are Modulated in Response to Curcuminoid Treatment

**Figure 2:**
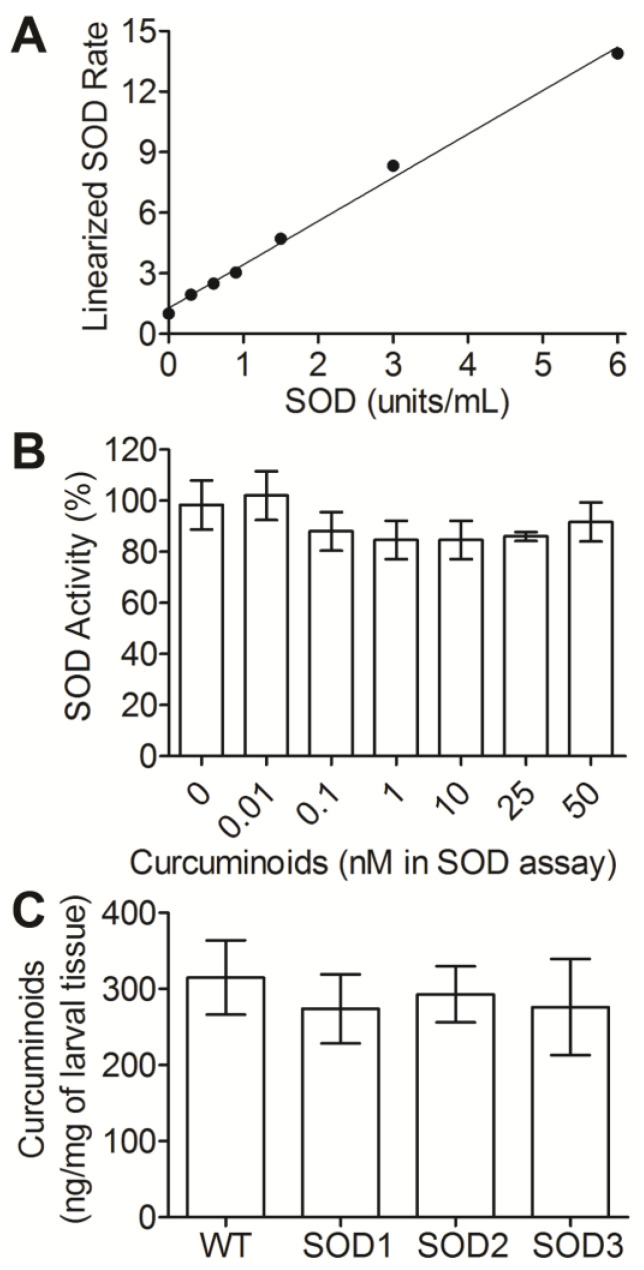
Parameters of the SOD activity assay used herein. A standard curve was prepared with purified SOD (**A**) with an equation of best fit: y=2.156∗X+1.274, (r^2^ = 0.9945). The addition of varying concentrations of curcuminoids to the SOD assay, conducted with a constant level of 3 units/mL SOD, is expressed as a percentage of the activity values recorded without the addition of curcuminoids (**B**). Each of three replicates was conducted in technical duplicate, averaged, and the mean of experiments conducted in triplicate is presented (**A**,**B**). Larvae from all strains were reared for seven days on 25 mM curcuminoids and the level of curcuminoids in tissues was quantified from groups of 10 crawling third-instar larvae (**C**) (*n* = 10 per treatment). The mean of experiments conducted in *n* = 10 is presented. Error bars in all panels represent the standard error of the mean. No significant differences were found in the data presented in panels (**B**,**C**).

Previous research has indicated that dietary curcumin decreases oxidative stress levels and leads to increased longevity [32,33]. Decreases in the levels of oxidative stress markers coincide with an increase in SOD expression, which would directly influence metrics of oxidative stress. While the link between dietary curcumin exposure and increased SOD expression has been demonstrated, the temporal dynamics of this interaction remain unexplored. To address this, a commercial SOD assay was used to measure SOD activity following chronic curcuminoid exposure and the addition or removal of curcuminoids from larval diets (Figure 2A). Following protein extraction from tubes of five washed and homogenized larvae (constituting a single biological replicate), it was found that 1 μg of total protein was sufficient to provide reproducible data. SOD activity in total protein from larvae reared without curcuminoids was measured immediately after extraction (0.79 ± 0.26 units/μg of protein) and following incubation on ice for one hour (0.66 ± 0.27 units/μg of protein). This duration on ice was within the time frame in which SOD activity was measured after extraction, and the additional time did not significantly alter SOD activity in samples (*t*-test, *p* = 0.7214). The SOD assay relies on the generation of superoxides by xanthine/xanthine oxidase for conversion into hydrogen peroxide and water by SOD; this reaction could be susceptible to the additional radical scavenging activity of curcuminoids present in larval tissue. Larvae reared in 25 mM curcuminoids were extracted with protein extraction buffer and HPLC analysis showed that extracted curcuminoids were below the limit of detection (1 ng on column or 45 nM). The addition of 0.01–50 nM curcuminoids to SOD assays using pure SOD supplied with the SOD assay kit did not result in significant decreases in activity at any concentration tested (Figure 2B; pairwise *t*-test, *p* > 0.07). The optimization steps described above provided the basis for the reproducible quantification of SOD activity in larval total protein extracts.

**Figure 3:**
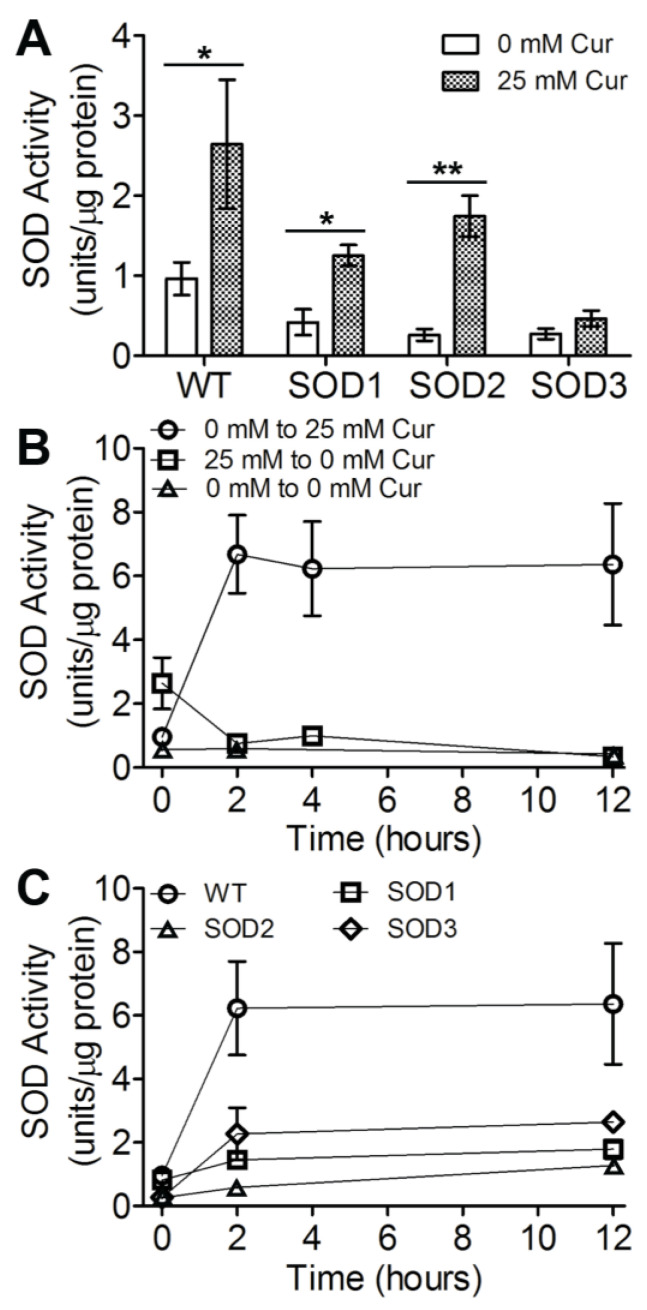
The influence of chronic and acute exposure to curcuminoids on SOD activity in the wild type (WT) and three SOD mutants. Larvae from all strains were reared for seven days on 0 or 25 mM curcuminoids; total protein was extracted, and SOD activity was quantified from groups of 5 crawling third-instar larvae (**A**) (*n* = 5 per treatment). WT larvae were reared for seven days on 0 or 25 mM curcuminoids, transferred to 25 mM or 0 mM (the opposite condition) for 2–12 h, total protein was extracted, and SOD activity was quantified from groups of 5 crawling third-instar larvae (**B**) (*n* = 5 per treatment). Larvae from all strains were reared for seven days on 0 curcuminoids, transferred to 25 mM for 2–12 h, total protein extracted, and SOD activity was quantified from groups of 5 crawling third-instar larvae (**C**) (*n* = 5 per treatment). The mean of experiments conducted in *n* = 5 is presented, and error bars represent the standard error of the mean; * *p* < 0.05, ** *p* < 0.005.

Studies have indicated that *Drosophila* reared on curcumin exhibit increased levels of SOD activity [9,22]. Quantification of the SOD activity of larvae reared on 25 mM curcuminoids (2.6 ± 0.9 units/μg of protein) displayed a 2.7-fold higher average SOD activity than larvae reared without curcuminoid supplementation (Figure 3A; 1.0 ± 0.2 units/μg of protein). In these experiments, SOD activity is measured after chronic exposure to curcumin; however, the influence of acute curcuminoid exposure or the removal of curcuminoids on SOD activity remained to be investigated. To determine the activity of SOD in response to acute curcuminoid exposure or the removal of curcuminoids, larvae reared on 0 or 25 mM curcuminoids were transferred to the opposite condition as described above, and SOD activity was quantified over the following 12 h. Larvae reared on 0 mM curcuminoids (1.0 ± 0.2 units/μg of protein) and transferred to 25 mM curcuminoids had a roughly 6.9-fold increase in average SOD activity after 2 h of exposure (6.7 ± 1.4 units/μg of protein). This level of SOD activity was consistent up to 12 h of exposure (Figure 3B). Interestingly, the SOD activity level measured following acute curcuminoid exposure is 2.4-fold higher on average than the level observed following seven days of chronic exposure to curcuminoids. In a similar trend, larvae reared on 25 mM curcuminoids and transferred to 0 mM curcuminoids saw a roughly 3.5-fold decrease in average SOD activity 2 h after transfer, which returned to the baseline level found in larvae raised on 0 mM curcuminoids. This level of SOD activity remained up to 12 h of exposure. While it is possible that the act of transferring larvae would be stressful and result in increases in SOD activity, transferring larvae from 0 mM curcuminoids to new medium with 0 mM curcuminoids did not alter SOD activity measurably. Taken together, the results indicate that the modulation of SOD activity occurs concomitantly with fluctuating curcuminoid levels. We infer that the changes in tissue curcuminoid levels directly or indirectly influence the mirrored trends in SOD activities.

### 3.3. Three SOD Genes Differentially Contribute to Curcuminoid-Induced Activity

Previous work has identified three distinct genes encoding SOD homologs in *Drosophila*, each with differing localization profiles in the cell. Research on mechanisms underlying cellular responses to curcumin in *Drosophila* has investigated SOD activity in total protein but has not assessed the contribution of the individual *SOD* genes. To determine the individual influence of each of the three *Drosophila SOD* genes in curcuminoid-induced SOD activity, the SOD activity in lines mutant for each of the three *SOD* genes was assessed following chronic or acute curcuminoid exposure as conducted above. Consistent with published results, each SOD mutant strain reared on GF medium without curcuminoid supplementation displayed decreased SOD activity compared to the WT (Figure 3A). Rearing the mutant strains on 25 mM curcuminoids for seven days resulted in increased SOD activity from all three strains compared to the 0 mM curcuminoid treatment. Only the SOD2 mutant approached WT activity levels on 25 mM curcuminoids while the other mutants displayed lowered SOD activities. While it is possible that the multiple mutations in the SOD mutant lines tested could impact curcuminoid accumulation in tissues, curcuminoid levels in larvae reared on 25 mM curcuminoids were not significantly different from the WT (Figure 2C; pairwise *t*-tests, *p* > 0.1). These results indicate that 25 mM curcuminoid exposure increases SOD activity, and that the three *SOD* genes exhibit different levels of involvement in this response, with SOD3 being the most active in this treatment.

In the wild type data presented above, it became clear that SOD activity was greater during acute curcuminoid exposures than following chronic exposure. It is therefore possible that the results of chronic curcuminoid exposure result in diminished SOD levels in the three *SOD* mutants compared to acute treatments. To determine whether an increased SOD activity level was observed in response to acute curcuminoid exposure, larvae from each of the three *SOD* mutants were transferred to media with 25 mM curcuminoids, and SOD activity was measured up to 12 h post transfer (Figure 3C). Like the wild type, an increase in SOD activity was evident 2 h post transfer; however, while the *SOD1* and *SOD3* mutants attain their maximal SOD level 2 h post transfer, the *SOD2* mutant sees an increase in SOD activity up to 12 h post transfer. The SOD activity of the *SOD1* and *SOD3* mutants 12 h post transfer was higher than that observed following chronic curcuminoid exposure (*t*-test, *p* < 0.05). In contrast, the SOD activity of the *SOD2* mutant was not significantly different between the acute and chronic treatments (*t*-test, *p* > 0.05). These results indicate that each SOD gene is involved in the response to curcuminoid treatment, but the dynamics of this response differ among genes and between acute and chronic exposures.

## 4. Discussion

In this study, we tested the hypothesis that the antioxidant role of dietary curcuminoids involves increases in SOD activity. We demonstrated saturation in curcuminoid treatments beginning at about 15 mM in our model, and that there is rapid uptake and clearance of curcuminoids in *Drosophila* larvae exposed to or removed from curcuminoid-supplemented medium. We additionally found that both acute and chronic curcuminoid treatment results in increased total SOD activity, and differences in SOD activity were observed between acute and chronic treatments. We found that mutants of each of the three *SOD* gene homologs displayed lower total SOD activity at baseline, and that this level increases following treatment with curcuminoids. This study indicates that each *SOD* gene is involved in the antioxidant response to curcuminoid treatment and highlights the differences in homolog involvement.

While recent studies have demonstrated that curcumin treatments are associated with increased SOD levels in some models, most report on only one of the isoforms or on the total SOD response [7,8]. To determine the contribution of various SOD isoforms to the curcuminoid-induced SOD activity, three different SOD mutant lines were exposed to curcuminoids in both chronic and acute regiments. The three isoforms differ in cellular localization and transcriptional regulators [2]. Interestingly, all three deficiency lines showed less induction of SOD activity by curcuminoids than the wild type in the acute regimen, but none were significantly different from the other SOD deficiency lines. The SOD2 deficiency line demonstrated more SOD activity induction by curcuminoids in the chronic regimen compared to the other deficiency lines (Figure 3A), which may result from the heterozygosity of the SOD2 [31,34].

The specific mechanism of action by which curcuminoids influence SOD activity remains unknown, and research has shown that many genes are differentially regulated in response to curcumin treatment [35]. Interestingly, studies in cell lines and other model systems found that curcumin, delivered in media or in diets, altered the expression of many genes in multiple pathways (e.g., SOD, catalase, interleukins, antimicrobial peptides, growth factors, etc.), particularly via the activities of NF-κB and the nuclear factor erythroid 2-related factor 2 (Nrf2) regulator [7,36,37]. Nrf2 has been shown to regulate the expression of many genes involved in the response to oxidative stress, including SOD [38,39]. Our findings indicate that SOD activity responds to curcuminoid levels within two hours of acute treatments, which is a relatively quick response if it is mediated through changes in the expression of Nrf2 and/or other genes. Previous work on responses to various physiological changes and stress indicate that sweeping changes in gene expression have been recorded 10–60 min following treatment onset [40,41,42]. Given that SOD activity is an important component of the flies’ response to acute oxidative stress, having a relatively quick response to treatment would seem beneficial for this organism.

Two different regimes of curcuminoid uptake were described in this work: a chronic condition where the larvae had curcuminoids in their diet throughout development and an acute condition where curcuminoids were only available for up to 12 h of feeding. Larvae, following chronic exposure, retain roughly three times more curcuminoids than larvae in the acute condition (Figure 1). Additionally, larvae reared in the chronic exposure treatment regimen initiated curcuminoid exposure as first-instar larvae, while those with acute exposure initiated treatment as third-instar larvae, which may underlie the observed differences in magnitude of SOD activity between the two exposure regimens. While the 25 mM concentration of curcuminoids is directly comparable between conditions, the developmental phase(s) were quite different. It is possible that curcuminoid uptake in first- and second-instar larvae is different than in third-instar larvae; the number of days of feeding somehow facilitates greater storage of curcuminoids. It is presumed that the curcuminoids are degraded at some rate within the larvae, so it is possible that degradation rates could be lower in earlier instar stages to allow greater levels of curcuminoids to accumulate. When larvae from the chronic curcuminoid condition were transferred to medium-lacking curcuminoids to assess clearance, some level of curcuminoids was retained within the larvae throughout the duration of the experiment. As the gut contents were allowed to clear for an hour prior to extraction, it is unlikely that this represented a large amount of the curcuminoids retained by larvae. It is possible that the first few hours of swift curcuminoid loss were due to clearance by the Malpighian tubules and the remaining curcuminoids were retained in fat globules and other tissues. While the above suggestions on possible underlying causes for the observed uptake and release of curcuminoids from larvae are strictly speculation, they will be the subject of future experiments.

## 5. Conclusions

In this work, the dynamics of curcuminoid uptake and clearance were assessed in *Drosophila* larvae and alterations in the level of these compounds appeared to mirror changes in SOD activity. Each of the three *SOD* gene homologs contributed to the curcuminoid-induced observed changes in SOD activity. As SOD activity is generally presented as total activity measurements without consideration of the influence of each *SOD* gene, this work provides insights into differential contributions to activity in response to curcuminoids. Presenting the initial measurements of dietary curcuminoid uptake and clearance and assessing the corresponding changes in SOD activity provide a general framework for consideration of the interplay of antioxidants and oxidative stress responses in this system.

## Data Availability

The data used in this work are presented in their entirety.
